# Metabolic Engineering Strategies of Industrial Hemp (*Cannabis sativa* L.): A Brief Review of the Advances and Challenges

**DOI:** 10.3389/fpls.2020.580621

**Published:** 2020-12-08

**Authors:** Michihito Deguchi, Shriya Kane, Shobha Potlakayala, Hannah George, Renata Proano, Vijay Sheri, Wayne R. Curtis, Sairam Rudrabhatla

**Affiliations:** ^1^The Central Pennsylvania Research and Teaching Laboratory for Biofuels, Penn State Harrisburg, Middletown, PA, United States; ^2^School of Medicine, Georgetown University, Washington, DC, United States; ^3^Department of Chemical Engineering, The Pennsylvania State University, University Park, PA, United States

**Keywords:** cannabinoid, CRISPR/Cas, *Cannabis sativa*, metabolic engineering, RNA interference

## Abstract

Industrial hemp (*Cannabis sativa* L.) is a diploid (2*n* = 20), dioecious plant that is grown for fiber, seed, and oil. Recently, there has been a renewed interest in this crop because of its panoply of cannabinoids, terpenes, and other phenolic compounds. Specifically, hemp contains terpenophenolic compounds such as cannabidiol (CBD) and cannabigerol (CBG), which act on cannabinoid receptors and positively regulate various human metabolic, immunological, and physiological functions. CBD and CBG have an effect on the cytokine metabolism, which has led to the examination of cannabinoids on the treatment of viral diseases, including COVID-19. Based on genomic, transcriptomic, and metabolomic studies, several synthetic pathways of hemp secondary metabolite production have been elucidated. Nevertheless, there are few reports on hemp metabolic engineering despite obvious impact on scientific and industrial sectors.

In this article, recent status and current perspectives on hemp metabolic engineering are reviewed. Three distinct approaches to expedite phytochemical yield are discussed. Special emphasis has been placed on transgenic and transient gene delivery systems, which are critical for successful metabolic engineering of hemp. The advent of new tools in synthetic biology, particularly the CRISPR/Cas systems, enables environment-friendly metabolic engineering to increase the production of desirable hemp phytochemicals while eliminating the psychoactive compounds, such as tetrahydrocannabinol (THC).

## Introduction

There is evidence of the historical use of industrial hemp (*Cannabis sativa* L.) in human civilization for both its phytochemical and lignocellulosic biomass properties. Hemp’s native origin appears to be Eurasia with distribution around the world primarily as a fiber crop (Frassinetti et al., 2018). The emergence of petrochemical-derived polymer fiber sources decreased the demand for hemp; however, its use as a food and feed supplement has increased because it contains essential fatty acids (*omega*-6 and *omega*-3), easily digestible proteins (albumin and edestin) and enhanced levels of the amino acid arginine, which has indications for cardiovascular health (Bonini et al., 2018). Recently, more attention has been given to its rich repertoire of pharmaceutical compounds (Izzo et al., 2009; Degenhardt et al., 2017).

To date, more than 540 phytochemicals have been reported in hemp (Andre et al., 2016). Of these, cannabidiol (CBD) is generally most abundant and promising phytochemical as it has shown potential as a therapeutic agent in preclinical models of central nervous system diseases (Hill et al., 2012). Unlike tetrahydrocannabinol (THC), which has been associated with numerous side effects (Russo, 2011), CBD has an extremely safe profile in humans (Pertwee, 2008; Zuardi, 2008). Recently, the FDA has approved CBD (epidiolex) as an anticonvulsant drug (Brown and Winterstein, 2019). Additionally, hemp produces other cannabinoids and terpenes that exhibit a wide array of pharmacological properties (McPartland and Russo, 2001; Izzo et al., 2009; Russo, 2011). Since there is an increased demand for hemp-derived medicinal products, it is imperative to adapt biotechnological methodologies to generate new hemp strains with significant quantities of phytochemicals of medical interest.

**Figure 1:**
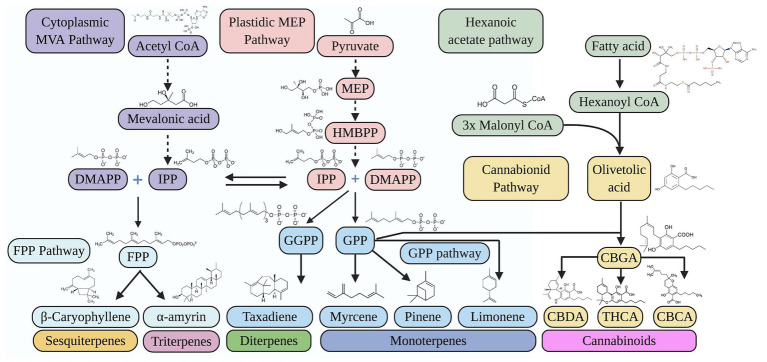
Biosynthetic pathways for cannabinoids and terpenoids in hemp. CBCA, cannabichromenic acid; CBDA, cannabidiolic acid; CBGA, cannabigerolic acid; DMAPP, dimethylallyl diphosphate; FPP, farnesyl diphosphate; GGPP, geranylgeranyl pyrophosphate; GPP, geranyl diphosphate; HMBPP, (E)-4-hydroxy-3-methyl-but-2-enyl pyrophosphate; IPP, isopentenyl diphosphate; MEP, methylerythritol phosphate; MVA, mevalonate; THCA, tetrahydrocannabinolic acid. Created with BioRender.

Synthetic pathways for representative cannabinoids and terpenes have been elucidated (Figure 1), but the metabolic engineering of the pathway genes, enzymes, and metabolite regulation remains to be studied (Bonini et al., 2018). Thus, the development of an efficient regeneration and stable transformation system is essential. In this review, we present the strategies of target gene selection for hemp metabolic engineering. Challenges and opportunities to utilize transient and stable gene expression approaches are also discussed toward achieving a reliable metabolic engineering system in hemp.

## Strategies of Target Gene Selection for Enhancing Phytochemical Yield in Hemp

Manipulation of single that genes encode biosynthetic enzymes attempt to target genes that regulate the supply of precursors for the synthetic pathway that usually regulate flux into the pathway (targeting precursor-synthesizing gene), or rate-limiting step enzyme coding genes (targeting phytochemical pathway gene). Genetic engineering now permits more explicit manipulation of these metabolic fluxes (O’Connor, 2015), where we suggest that the native *Cannabis* host represents an ideal platform for this commercialization that is currently limited by the limitations of applicability of current biotechnological tools.

### Targeting Phytochemical Pathway Gene

A focus on rate-limiting step is particularly effective for demonstrating function, as it permits confirming biochemistry in the absence of a background. This is exemplified in work of Sirikantaramas et al. (2004) who introduced the tetrahydrocannabinolic acid synthase (THCAS) gene in tobacco to synthesize THCA from cannabigerolic acid (CBGA), which was exogenously through roots. With this strategy, the composition of cannabinoids can be modified to more desirable such as high cannabidiolic acid (CBDA) or CBGA contents by overexpression of *CBDA synthase* (*CBDAS*) or *aromatic prenyl transferase/PT4*, respectively. Knockout of THCA by silencing *THCA synthase* (*THCAS*) will be expected, given the fact that other plant-derived prenyl-compounds are difficult to isolate from psychoactive THCA due to the structural similarity (Valliere et al., 2019). The quantity of CBGA will also be increased by simultaneous silencing of *THCAS* and *CBDAS*. Besides, silencing of both *THCAS* and *CBDAS* will expedite the production of minor cannabinoid, cannabichromenic acid (CBCA) which is difficult and expensive for pharmacological studies in clinical trials because of low abundance (Gülck and Møller, 2020).

### Targeting Precursor Synthesizing Gene

Enzyme capacity for metabolism would be reasonably matched to the substrate availability for a pathway. Therefore, the ability to increase product formation with targeting phytochemical pathway gene will logically be quickly limited by the metabolite flux of upstream precursors (Balcke et al., 2017). In this scenario, metabolic engineering to increase precursor of synthetic pathway has been demonstrated to progressively increase downstream products (Ku et al., 2020). For the engineering of cannabinoid synthesis genes, the supply of the high amount of geranyldiphosphate (GPP), which is a substrate to synthesize CBGA is essential. GPP is produced from isopentenyl diphosphate (IPP) in plastid (Figure 1), thus the overexpression of GPP synthase or activating MEP-pathway genes that resulted in higher IPP concentration in the plastid will be useful (Schachtsiek et al., 2018). Olivetolic acid, which is other substrate for CBGA synthesis, is formed from hexanoyl CoA by both tetraketide synthase and olivetolic acid cyclase. According to Stout et al. (2012), the concentration of hexanoyl-CoA paralleled the accumulation of the CBDA, which indicates that the synthesis of hexanoyl-CoA will be rate-limiting step in cannabinoid biosynthesis. Thus, overexpression of two isoforms for acyl-activating enzyme (AAE) 1 and 3 will lead to increase the supply of hexanoyl-CoA. Importantly, comprehensive gene expression was studied in nine *Cannabis* strains with different phytochemical content, which revealed crosstalk between cannabinoid and terpene accumulation (Zager et al., 2019). Further study will provide an insight to understand which precursors need to be supplied in excess to increase the synthesis and accumulation of target metabolites.

### Pathway Activation

Unlike microbial metabolite pathways that are often polycistronic, the genes for plant secondary metabolites are scattered throughout the vast plant genomes. The manipulation of genes that are linked though transcription factors provides a means to upregulate a pathway, but circumventing the signal transduction that precedes coordinated regulation such as a pathogen defense response. This strategy has been applied to many plant secondary metabolites, including anthocyanins in *Arabidopsis* (Liu et al., 2018; Outchkourov et al., 2018), flavonoids in tomato (Stracke et al., 2007; Luo et al., 2008), and alkaloids in *Catharanthus roseus* (Van Moerkercke et al., 2015; Pan et al., 2019) by upregulation of transcription factors. In *Cannabis*, van Bakel et al. (2011) identified several dozen transcription factors that are likely to play roles in the regulation of the THC synthesis pathway. Additionally, Marks et al. (2009) demonstrated the function of two MYB-domain transcription factor that seem to regulate the cannabinoid synthesis in the *Cannabis* trichome. These transcription factors are targets to activate cannabinoid synthesis. Notably, cannabinoid synthesis is expedited by UV light application or heavy metal (Zhang and Björn, 2009; Husain et al., 2019). The elucidation of signal transduction triggered by these elicitors may lead to the discovery of positive and negative regulators of signal transduction, which will be the target genes for hemp metabolic engineering.

### Alternative Platforms for Hemp Phytochemical Production

The nature of metabolic engineering introduces the opportunity to not only examine a native production platform for a biochemical, but also the potential to move that biosynthesis into an alternative host. Microbial platforms have served as elegant platforms for the elucidation of plant metabolite function (Pyne et al., 2019), and will likely be extremely valuable in elucidating yet unknown enzymatic conversions in hemp cannabinoids. For higher value, and immediate market-driven production, platforms such as this can be expected to provide specific metabolites in hemp. Recently, CBDA was synthesized in yeast *via* the introduction of the MEP pathway, GPP pathway, hexanoic acetate pathway, and CBDA synthesis pathway (Zirpel et al., 2017; Luo et al., 2019). On the other hand, heterologous production in other plant species is still a challenge. It is not unusual for heterologous metabolite production platforms to lack physiological requirements for the high productivity that can be observed in native systems (Schachtsiek et al., 2018). In cannabinoid synthesis, toxicity effects must be considered, as several cannabinoid pathway metabolites such as CBGA and THCA cause cell death *via* apoptosis in host plant (Sirikantaramas et al., 2005). In hemp, olivetolic acid synthesized in cytosol is transferred to plastid, where olivetolic acid and geranyl-PP are converted into CBGA, which is finally released to apoplast (Gülck and Møller, 2020). It will be critical to elucidate the mechanism underlying transport and accumulation of metabolites and apply it to better hemp phytochemical production in other plant species commercially (Table 1). Looking toward the future of hemp “designer lines” to produce various phytochemicals, we now focus the remainder of this review on the challenge of advancing biotechnological methods of plant transformation and regeneration as it applies to hemp.

**Table 1:** Companies who have utilized genetic modification techniques to produce cannabinoids on an industrial scale.

Company and location	Product detail
Canopy Growth Corp.; Smiths Falls, Canada	Largest legal *Cannabis* company in the world and has partnerships/acquired the following companies: Ebbu; Spectrum Therapeutics; Canopy Innovation Lab; Storz & Bickel; Ebba, Battelle, Apollo, and Scientus.
Ebbu; Evergreen, CO, United States	Developed CRISPR–Cas9 to produce plants that secrete only CBD and only CBG.
Zenabis; Vancouver, Canada	Sells both recreational and medical *Cannabis* and have the following subset companies: Vida, Zen Craft Grow, Namaste, Blazery, and Re-Up.
Farmako; Frankfurt, Germany	Turned to *Zymomonas mobilis* bacterium to make 180 cannabinoids, including THC and CBD to use in *Cannabis*-based drug therapies.
Ginkgo Bioworks; Boston, MA, United States	Synthetic-biology company that worked with the Croncos Group to manufacture pure CBD and other cannabinoids in yeast.
Croncos Group; Toronto, Canada	Focuses on advancing *Cannabis* research, technology, and product development. Has a brand portfolio that includes PEACE NATURALS, COVE, SPINACH, Lord Jones and PEACE+.
Librede; Carlsbad, CA, United States	Synthetic-biology company with patent to use yeast (*Saccharomyces cerevisiae*) to synthesizing cannabinoids from sugars while being sustainable.
Demetrix; Emeryville, CA, United States	Uses *Saccharomyces cerevisiae* controlled fermentation to produce rare cannabinoids.
Maku Technologies; Durham, North Carolina, United States	Focuses on producing rare, natural cannabinoids in yeast to increase research on cannabinoids.
InMed Pharamaceuticals; Vancouver, Canada	Produces enzymes with *Escherichia coli* biofermentation to yield cannabinoids through the process of biotransformation and other purification stages. The cannabinoids can also be converted to other rare cannabinoids.
Renew Biopharma; San Diego, CA, United States	Uses *Chlamydomonas reinhardtii* to produce cannabinoids and uses the cannabinoids to target certain receptors that contribute to brain inflammation and chronic pain. Has a patent for the NphB enzyme in cannabinoid synthesis.
Teewinot Life Sciences; Tampa, Florida, United States	Has a patent for a bioreactor designed to grow cannabinoid-producing microorganisms called CannSynthesis. Can produce 25 minor cannabinoids and are developing a library of cannabinoid analogs.
Trait Biosciences Toronto, Canada	Identified a gene that when expressed in *Cannabis* leads to increased trichome production and upregulation of cannabinoids. Creates water-soluble cannabinoids, customizes cannabinoid profiles, and produces THC-free hemp.
InPlanta Biotechnology; Lethbridge, Canada	Focuses on growing *Cannabis* with specific CBD/THC/terpenoid contents and breeding high CBD hemp.
Dewey Scientific; Pullman, WA, United States	Offers scientific insights to *Cannabis* producers to increase efficiencies and crop yields while decreasing crop inputs by looking at molecular biology and traditional breeding.

### Transgenic Gene Delivery Systems

#### Tissue Culture and Stable Transformation

To establish an efficient transformation system, the development of a hemp regeneration protocol is critical. Until recently, a variety of explants such as leaf, hypocotyl, cotyledon, stem, axillary bud, petioles, and shoot tips were tested with the combination of different auxins and cytokinins for the purpose of direct or indirect regeneration (Table 2). Lata et al. (2009a) demonstrated the induction of high-frequency shoot regeneration from nodal segments containing axillary buds using thidiazuron (TDZ). Lata et al. (2010) obtained the highest shoot induction rate at 0.5 μM TDZ in callus, whereas Chaohua et al. (2016) demonstrated the highest shoot induction at 2.0 μM TDZ in the cotyledon. Wielgus et al. (2008) tested various combinations of plant growth regulators and obtained regenerated plants on MS medium containing benzoic acid herbicide: DICAMBA. A cytokinin meta-topolin was also effective for shoot regeneration from nodal explants (Lata et al., 2016a). *In vitro* propagation has also been studied in *C. sativa* and reviewed by Lata et al. (2017). Recently, Kodym and Leeb (2019) established a photo-autotropic micropropagation system and obtained a 97.5% rooting rate from the *in vitro* generated shoot tip cuttings.

**Table 2:** Overview of previously reported tissue culture and stable transformation work in *Cannabis sativa*.

Explant	Variety	Reference
Seedlings	Unknown	Veliky and Genest, 1972
Root, hypocotyl, leaves, male, and female floral parts	Unknown	Itokawa et al., 1975
Leaves, bracts, anthers, and maturing leaves	Mexican drug-type (152) and Turkish fiber-type (150)	Hemphill et al., 1978
Epicotyls	*C. sativa* var. indica	Heitrich and Binder, 1982
Embryo, leaf, and stem	Unknown	Loh et al., 1983
Seedlings	Unknown	Hartsel et al., 1983
Stem, cotyledon, and root	Unknown	Fisse and Andres, 1985
Leaf	THC dominant strain from South Africa	Braemer and Paris, 1987
Leaf	Carmagnola, Fibranova, Uniko, and Kompolti	Mandolino and Ranalli, 1999
Seedlings	Fedora 19 and Felina 34	Mackinnon et al., 2000
Stem and leaves	Anka, Uniko-B, Felina-34, and Kompolti	Feeney and Punja, 2003
Internodes, axillary buds, and petioles	Silesia, Fibrimon-24, Novosadska, Juso-15, and Fedrina-74	Slusarkiewicz-Jarzina et al., 2005
Roots, leaves, and stem	Beniko, Bialobrzeskie, and Silesia	Plawuszewski et al., 2006
Leaves, flowers, and 4 days old seedlings	Four-Way	Raharjo et al., 2010
Cotyledon, stem, and root	Bialobrzeskie, Beniko, and Silesia	Wielgus et al., 2008
Nodal segments containing axillary buds	MX-1	Lata et al., 2009a
Nodal segments with axillary buds	MX-1	Lata et al., 2009b
Shoot tips	Changtu	Wang et al., 2009
Leaf	Skunk	Flores-Sanchez et al., 2009
Leaf	MX-1	Lata et al., 2010
Nodal segments with an axillary bud	MX-1	Lata et al., 2012
Hypocotyl	Futura77, Delta-llosa, Delta405, CAN0111, and CAN0221	Wahby et al., 2013
Cotyledon and Epicotyl	Iranian *Cannabis*	Movahedi et al., 2015
Internodes	Long-ma No.1	Jiang et al., 2015
Stem and leaves	Anka	Feeney and Punja, 2015
Leaf and hypocotyl	Iranian *Cannabis*	Movahedi et al., 2016a
Leaf and hypocotyl	Iranian *Cannabis*	Movahedi et al., 2016b
Cotyledons	Dioecious hemp from Changsha, China	Chaohua et al., 2016
Nodal segments with axillary buds	MX-1	Lata et al., 2016a
Nodal segments	MX-1	Lata et al., 2016b
Hypocotyl, cotyledons, and leaves	Futura77, Delta-llosa, and Delta405	Wahby et al., 2017
Leaf	Canda, Joey, Landrace, Futura, and CFX-2 (Cherry × Workhorse)	Thacker et al., 2018
Seedlings	Futura	Gabotti et al., 2019
Hypocotyl segments	Bialobriezskie, Tygra, Fibrol, Monoica, and USO-31	Smýkalová et al., 2019
Hypocotyl	Ferimon, Felina32, Fedora17, USO31, and Finola	Galán-Ávila et al., 2020
Seedlings	BA-1, BA-2, BA-41, BA-49, BA-61, and BA-71	Page et al., 2020
Leaf	GRC, RTG, U22, U31, U37, U38, U42, U61, U82, and U91	Monthony et al., 2020
Nodal and tip cuttings	Epsilon 68	Wróbel et al., 2020

Subsequently, a few successful *Cannabis* transformation systems were reported by Lata et al. (2017). Suspension cell culture and hairy roots were transformed *via Agrobacterium*, whereas the transformed tissues were not regenerated (Feeney and Punja, 2003). Hypocotyl was inoculated with *Agrobacterium*, and the shoot was produced in MS media containing 6-benzylaminopurine and zeatin, which resulted in complete hemp transgenic plant (Sirkowski, 2012, United States Patent application 20120311744A1). Nevertheless, *Cannabis* regeneration and stable transformation are still limited to specific varieties, and a reliable transformation protocol has not been established (Salentijn et al., 2019), partially because of limited breeding that could establish homogenous lines.

**Figure 2:**
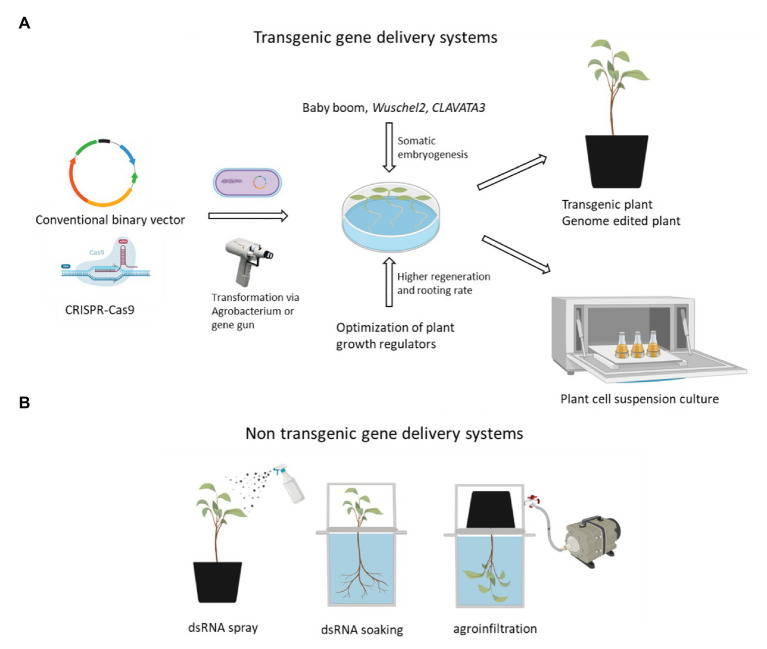
A schematic strategy of transgenic and transient gene delivery system. These gene delivery systems aim to alter gene expression in hemp female flower, where cannabinoids and terpenes are preferentially synthesized and stored. **(A)** Transgenic gene delivery systems. Specialized metabolites and their precursor synthesis genes, transcription factor genes, and other hemp genes related to the supply of energy and reducing power might be engineered by introducing conventional binary vectors or CRISPR/Cas vectors. Hemp SE might be activated by the overexpression of positive regulator genes such as *BBM* and *WUS2* or the downregulation of negative regulator genes such as *CLAVATA3*. **(B)** Three transient gene delivery systems. To overcome the instability of dsRNA, clay nanoparticles, liposomes, viruses, or bacteria might be used for the effective delivery of dsRNA. For successful agroinfiltration, vacuum infiltration is likely to be more efficient than syringe infiltration based on our preliminary experiments. Created with BioRender. CRISPR/Cas-mediated genome editing is likely to be most powerful method for hemp metabolic engineering.

It is worth highlighting that plant mechanism underlying somatic embryogenesis (SE) and subsequent regeneration system has been elucidated, and key regulators of plant cell totipotency were identified (Figure 2; Horstman et al., 2017; Méndez-Hernández et al., 2019). Upregulation of morphologic regulator (MR) genes has promoted regeneration rate in both monocotyledonous species (Lowe et al., 2016; Mookkan et al., 2017; Hoerster et al., 2020) and dicotyledonous species (Deng et al., 2009; El Ouakfaoui et al., 2010; Florez et al., 2015). The alteration of gene expression on hemp MR would open the door to enhance SE and facilitate the acquisition of regenerated and transformed hemp plants and developing a synthetic seed technology for commercialization of hemp clones.

#### Genome-Editing Technologies

The CRISPR-Cas system currently emerged as a genome editing tool with the simplicity of target design, high efficiency of editing, multiplex knock-in/out ability, and low cost (Jaganathan et al., 2018). In plant metabolic engineering, CRISPR/Cas-mediated engineering is robust and convenient to generate knockouts of target genes *via* the DNA repair pathway: nonhomologous end-joining (NHEJ). Because of its ability to perform simultaneous gene knockouts, this system can be used to eliminate undesired multiple branching pathways (Alagoz et al., 2016; Sun et al., 2017). Recent advances in multiplex genome-editing tools have made it possible to delete six tomato genes by expressing 12 gRNAs *via* the expression of a single CRISPR vector (Čermák and Curtin, 2017). This multiplex mutation system will be highly useful for hemp metabolic engineering too, for instance, simultaneous knocking down of *THCAS* and *CBDAS* to increase the synthesis of minor cannabinoids with potential pharmaceutical values that are present in hemp but have not been studied because of their low quantity.

Although many CRISPR tools are most effective for the knockout of endogenous genes, the overexpression of endogenous genes *via* homology-directed repair (HDR) is still a challenge in many plant species (Hahn et al., 2018). Targeting a cis-regulatory element is thus a viable alternative. A cis-regulatory element is a noncoding DNA region that contains binding sites for transcription factors or other proteins that control transcription. Recent research has demonstrated the enormous potential of editing cis-regulatory elements to regulate optimal gene expression denominated as “fine-tuning” (Shrestha et al., 2018; Wolter et al., 2019). Moreover, editing untranslated region (UTR) is also a useful approach for fine-tuning genes of interests given the fact that UTRs play an important role in the regulation of protein synthesis (Si et al., 2020).

Base editing has emerged as a newly developed technique that enables direct, irreversible conversion of one base pair to another without disruption of a gene or requiring a donor template (Mishra et al., 2020). Base editors such as cytosine base editor and adenine base editor are basically composed of cytosine or adenosine deaminase domain, respectively, and catalytically inactive CRISPR–Cas9 domain (Kang et al., 2018). The base-editing system can generate a single-base change or single nucleotide polymorphisms (SNP), thereby facilitating plant breeding and basic research (Monsur et al., 2020). In *Cannabis*, more than 14,000 SNPs were genotyped in both drug type and fiber type strains (Sawler et al., 2015). Among them, several SNPs that are associated with Cannabinoid contents have been identified (Rotherham and Harbison, 2011; Onofri et al., 2015; Borna et al., 2017). These SNPs can be good targets for hemp metabolic engineering *via* base editing with the aim of altering the property of cannabinoid content.

It is important to note that these three editing methods: CRISPR/Cas mediated gene mutation *via* NHEJ, fine-tuning of gene expression *via* cis-regulatory elements, and base editing follow a cisgenic approach (Holme et al., 2013; Hou et al., 2014) and therefore, do not introduce any exogenous genes. Consequently, it is easier to obtain public acceptance for commercializing hemp products obtained *via* these methods, especially for hemp consumers that are usually averse to products obtained from genetically modified plants (Schluttenhofer and Yuan, 2017).

Of the three methods discussed, the CRISPR/Cas9 system shows the broadest utility, and it would be very beneficial to introduce CRISPR/Cas to hemp metabolic engineering for the following four reasons:

1. Hemp is a diverse and polymorphic species (Weiblen et al., 2015), and due to genome duplication, the gene copy number is high on many of hemp genes including phytochemical synthesis genes (van Bakel et al., 2011), which require more studies to identify functional genes. This makes genetic engineering more complex in this plant. On the contrary, CRISPR/Cas9 enables the knockout of several homologous genes *via* a single editing step (Jacobs et al., 2017).
2. Most hemp varieties do not self-pollinate; it is not feasible to obtain homozygous plants by self-pollination. However, CRISPR makes it possible to mutate or modify the gene of interest in both alleles at one editing step such that homozygous plants can be obtained in T0 editing generation. Shen et al. (2017) demonstrated that high efficiencies of site-specific double-stranded breaks allowed the isolation of mutants carrying homozygous mutated alleles of eight targeted genes simultaneously in rice.
3. In hemp, there is no established protocol for mutagenesis (Salentijn et al., 2019). Bielecka et al. (2014) showed that isolation of mutants from chemical mutagenesis screen is possible, but extremely difficult due to the anemophilous and dioecious nature of hemp. Genome editing technology allows for very specific gene editing and makes it easy to evaluate the effect of off-target gene modification. Therefore, this technology provides precision that is not possible with mutation breeding such as EMC mediated mutation that resulted in hundreds of unexpected mutations (Henry et al., 2014). Furthermore, a reverse genetic approach using the CRISPR/Cas mediated mutants will drastically accelerate the study of the function of synthetic pathway genes of specialized metabolites as there is gene silencing tools such as VIGS in hemp are just started to be developed by Schachtsiek et al. (2019).
4. Lastly, CRISPR/Cas system will drastically shorten the hemp breeding time. The methods commonly used in hemp breeding are “mass selection,” “cross-breeding,” “inbreeding,” and “hybrid breeding” (Clarke and Merlin, 2016), and creating unique *Cannabis* strains *via* these traditional methods is time-consuming and takes both patience and persistence. Indeed, conventional *Cannabis* breeding has expanded to include the diverse composition of elite varieties, ranging from plants with no THCA to those with high concentrations of CBDA or terpenes (Russo, 2019). However, CRISPR/Cas-based editing system is able to carry out pyramiding multiple desirable traits such as phytochemical properties, degree of monoecy, length of vegetative cycle, and resistance to diseases and pest (Salentijn et al., 2019) in one editing step, which is not feasible or takes at least 10 years in conventional breeding (Schluttenhofer and Yuan, 2017).

### Transient Gene Delivery Systems

#### Transient Gene Expression *Via* Agroinfiltration

Agroinfiltration is a prominent methodology for temporarily expressing a gene of interest easily and rapidly. This technology was first explored in molecular studies, including transient reporter gene expression, promoter analysis, and protein-protein interactions (Norkunas et al., 2018). Optimization of agroinfiltration has become a technique to produce vaccines, enzymes for industrial use, and secondary metabolites (Lai and Chen, 2012; Chen and Lai, 2013; Gleba et al., 2014; Reed and Osbourn, 2018). Agroinfiltration thereby provides an alternative method for stable transformation (Andrews and Curtis, 2005; Chen and Lai, 2015). Nevertheless, strong transient gene expression achieved *via* agroinfiltration is limited to some model crops, and agroinfiltration protocols for many agronomically important crops have only recently been optimized (King et al., 2015). Most recently, Deguchi et al. (2020) optimized the proper concentration of surfactant and antioxidants for *Agrobacterium* vacuum infiltration and achieved gene overexpression and silencing in hemp trichome. Schachtsiek et al. (2019) inoculated *Agrobacterium* carrying VIGS-vectors to *Cannabis* mature leaf which led to the reduction of 70% of gene expression in phytoene desaturase and magnesium chelatase subunit I. Enhanced efficiency of transient expression will not only pave the way for metabolic engineering but will also contribute to successful *Agrobacterium* inoculation into explants for stable transformation.

#### Topical Application of dsRNA

RNA interference (RNAi) is a gene regulation mechanism that induces the silencing of gene expression at the transcriptional or posttranscriptional level in eukaryotes (Petrick et al., 2013). In plants, this silencing mechanism has been used to confer resistance against pests and diseases by genetic transformation (Koch and Kogel, 2014). More recently, the topical application of dsRNA has emerged as an alternative to the generation of genetically modified plants. A prerequisite for the success of this technology is the efficient delivery of dsRNA to the plant. There have been several methods developed for achieving this, but the two most useful approaches are the soaking of the plant root and spray application on the surface of the plant (Andrade and Hunter, 2016). The topical application of dsRNA can be designed and tested much faster than the stable transformation of plants, which makes these approaches suitable for recalcitrant plants to stable transformation like hemp. Furthermore, double-stranded RNAs are present naturally in plants and normally degraded within a few days, rendering this technology more environment-friendly than others (Wang et al., 2011). Therefore, it is apt for hemp consumers that demand organic products and environmental sustainability of the crop. To date, this transient gene silencing approach has been successfully applied to control insects, fungi, and viruses (Koch et al., 2016; Mamta and Rajam, 2017; Dalakouras et al., 2019).

Attempts to convert the topical application of dsRNA to phytochemical production represent a new challenge. There are some reports from private companies regarding the development of RNAi spray and soaking protocols for *Cannabis* metabolic engineering, but the details of this technology have not been disclosed. The challenging task of identifying important targets in secondary metabolite pathways has been overcome with bioinformatic information such as hemp genomic and transcriptomic sequences, and a global map of metabolic pathways provided by the Kyoto Encyclopedia of Genes and Genomes (KEGG; Kanehisa and Goto, 2000). The instability of the naked dsRNA applied on plants is another barrier, but this could be rectified by using clay nanoparticles, liposomes, viruses, or bacteria as potential dsRNA carriers for spray application to achieve a longer-term gene-silencing effect (Joga et al., 2016; Mitter et al., 2017). Even if dsRNA technology has limited field applicability due to economic considerations, it could provide an exceptional tool for plant improvement in experimental settings.

## Discussion

To date, production of improved hemp through the genetic engineering has been limited due to low shooting and rooting efficiency. However, newly emerging biotechniques are anticipated to overcome this barrier. The most powerful approach is likely to involve manipulation of MR to enhance the developmental pathway of somatic embryogenesis. Introduction of genes into the hemp genome does not appear to be limiting as for example the creation of “hairy roots;” however, the transition of that cell into a viable plant requires a major reprogramming of plant cell development. Using appropriate surfactant and antioxidant reagents, *Agrobacterium* infection to hemp explant became no longer a difficult step *via* vacuum infiltration (Deguchi et al., 2020). Once somatic embryogenesis is achieved by altering the transient expression of morphologic regulator genes, hemp explants would regenerate at a high rate and be available for further genetic modifications with value added traits. If highly efficient somatic cell conversion can be achieved, this technological advance can even be applied to rapid propagation of enhanced hemp phenotypes with some technologies now advancing to synthetic seeds. Interestingly, Maher et al. (2020) demonstrated that MRs worked compatibly with the CRISPR/Cas system in various dicot species.

Recently, hemp phytochemical pathway genes have been extensively studied *via* omics approaches (Braich et al., 2019; Vincent et al., 2019; Gao et al., 2020). However, the characterization of function on most of phytochemical pathway genes remains to be studied. Advancement of molecular biology tools and the establishment of a hemp transformation system will not only achieve a diverse of hemp new varieties with improved quality and/or quantity of phytochemicals but also further intensify the investigation of other minor cannabinoid and terpene synthesis genes as well as several representative cannabinoid synthesis genes to expand the pharmacological potential of the hemp biochemical production platform.

## Author Contributions

SR and MD designed the concept for the review paper. MD, SK, and SP organized and wrote the manuscript. SR, WC, VS, RP, and HG edited the manuscript. All authors contributed to the article and approved the submitted version.

### Conflict of Interest

The authors declare that the research was conducted in the absence of any commercial or financial relationships that could be construed as a potential conflict of interest.
